# Biochemical Markers in the Prediction of Pregnancy Outcome in Gestational Diabetes Mellitus

**DOI:** 10.3390/medicina60081250

**Published:** 2024-07-31

**Authors:** Vesna Mandić-Marković, Zorana Dobrijević, Dragana Robajac, Goran Miljuš, Miloš Šunderić, Ana Penezić, Olgica Nedić, Danijela Ardalić, Željko Miković, Ognjen Radojičić, Milica Mandić, Jelena Mitrović

**Affiliations:** 1 Faculty of Medicine, University of Belgrade, Dr Subotica 8, 11000 Belgrade, Serbia; mikovic.zeljko@gmail.com; 2 Department for High-Risk Pregnancies, University Clinic for Gynecology and Obstetrics “Narodni Front”, Kraljice Natalije 62, 11000 Belgrade, Serbia; danielaardalic@gmail.com (D.A.); ogi.radojicic@gmail.com (O.R.); milica96mandic@gmail.com (M.M.); jelenavugdelic@gmail.com (J.M.); 3 Department for Metabolism, Institute for the Application of Nuclear Energy, University of Belgrade, Banatska 31b, 11000 Belgrade, Serbia; zorana.dobrijevic@inep.co.rs (Z.D.); draganar@inep.co.rs (D.R.); goranm@inep.co.rs (G.M.); milos@inep.co.rs (M.Š.); anap@inep.co.rs (A.P.); olgica@inep.co.rs (O.N.)

**Keywords:** gestational diabetes mellitus, pregnancy outcome, biochemical markers, inflammation parameters, fibrinogen

## Abstract

*Background and Objectives*: Gestational diabetes mellitus (GDM) may impact both maternal and fetal/neonatal health. The identification of prognostic indicators for GDM may improve risk assessment and selection of patient for intensive monitoring. The aim of this study was to find potential predictors of adverse pregnancy outcome in GDM and normoglycemic patients by comparing the levels of different biochemical parameters and the values of blood cell count (BCC) between GDM and normoglycemic patients and between patients with adverse and good outcome. *Materials and Methods*: Prospective clinical study included 49 patients with GDM (study group) and 44 healthy pregnant women (control group) who underwent oral glucose tolerance test (OGTT) at gestational age of 24–28 weeks. At the time of OGTT peripheral blood was taken for the determination of glucose levels, insulin, glycated hemoglobin, lipid status, homeostatic model assessment, BCC, iron and zinc metabolism, liver function, kidney function and inflammatory status. Each group was divided into two subgroups—normal and poor pregnancy outcome. *Results*: Higher RBC, hemoglobin concentration, hematocrit value, fasting glucose, uric acid and fibrinogen were found in GDM patients compared to control group. In GDM patients with poor pregnancy outcome values of fibrinogen, ALT, sedimentation rate, granulocyte and total leukocyte counts were elevated, while the serum level of zinc was significantly lower. Higher level of fibrinogen was found in normoglycemic patients with adverse pregnancy outcomes. ROC curve was constructed in order to assess fibrinogen’s biomarker potential. The established AUC value for diagnostic ROC was 0.816 (*p* < 0.001, 95% CI 0.691–0.941), while the AUC value for assessing fibrinogen’s potential to predict poor pregnancy outcome in GDM was 0.751 (*p* = 0.0096, 95% CI 0.561–0.941). *Conclusions*: The results of our study demonstrated that the best prognostic potential in GDM showed inflammation related parameters, identifying fibrinogen as a parameter with both diagnostic and prognostic ability.

## 1. Introduction

Gestational diabetes mellitus (GDM), defined as hyperglycemia first recognized during pregnancy [1], is the most common medical complication during pregnancy. Incidence of GDM depends on diagnostic criteria and varies by the region, ranging from 9 to −25%, with a global incidence of 14% [2,3]. The rise in global incidence of GDM is a consequence of sedentary lifestyle, stress, high calory intake diet, obesity and rising maternal age, especially in urban population worldwide.

GDM may impact both maternal and fetal/neonatal/infant health causing short-term and long-term consequences. Short-term maternal outcome may be influenced by developing gestational hypertension or preeclampsia, polyhydramnios, premature rupture of membranes, induction of labor, increased incidence of Cesarean section, operative delivery, birth canal injuries and postpartum hemorrhage. Short-term fetal/neonatal consequences include preterm delivery, macrosomia, shoulder dystocia, neonatal hypoglycemia, jaundice, respiratory distress syndrome (RDS) and neonatal mortality [4]. Long-term impact on both maternal and infant health includes diabetes, obesity and cardiovascular disease [5,6].

Pathophysiology of GDM is complex, including pancreatic β-cell dysfunction; insulin resistance; gluconeogenesis; maternal and fetal hyperglycemia; enhanced oxidative stress; maternal gut microbiota dysfunction; epigenetic changes; and other, less defined, pathogenic mechanisms [7,8]. Maternal adipose tissue and the placenta produce specific factors that either play a role in the pathogenesis of GDM or are the result of an underlying etiological process that concurrently causes GDM. These factors, acting as potential biomarkers, may be indicators of GDM and the associated complications and their determination may be useful in prediction, early diagnosis, the prevention of progression and monitoring therapy of GDM. Commonly investigated biomarkers are metabolites, single-nucleotide polymorphisms (SNPs), microRNAs (miRNAs), and proteins [9,10,11,12]. Also, different biochemical parameters, such as blood count, glycemic and lipid status, uric acid, ferritin, transferrin and iron, along with microelements were investigated as predictors or risk factors of GDM especially during the first trimester [13,14]. However, none of these candidates has reached clinical application and the most promising indicators of glucose status, such as glycated hemoglobin, proved inefficient in predicting both GDM diagnosis and pregnancy outcome in GDM.

The determination of diagnostic and prognostic indicators of GDM in the second/early third trimester would be beneficial for improving GDM diagnosis and risk assessment, selection of women for diagnostic OGTT, and for patient monitoring and potential intervention. Therefore, the aim of this study was to identify potential valuable predictors of adverse pregnancy outcome in GDM and normoglycemic patients by comparing the levels of different biochemical parameters and the values of blood cell count between GDM and normoglycemic patients and between patients with adverse and good outcome in both groups.

## 2. Materials and Methods

A prospective observational clinical study was conducted at the University Clinic for Gynecology and Obstetrics “Narodni Front” (UCGO NF), Belgrade, Serbia and included 49 patients with GDM (study group) and 44 healthy pregnant women (control group). All pregnancies were singleton, with known gestational age, and without other pregnancy pathologies or previously diagnosed metabolic disease. Patients with gestational hypertension were excluded from the study. Inclusion and exclusion criteria are listed in Table 1.

The Ethics Committee of OGC NF granted institutional approval for the study (Approval No. 05006-2019-4925) in accordance with internationally accepted ethical standards (The Helsinki Declaration of 1964, as revised in 1975, 1983 and 1989) and each patient signed the informed consent form.

**Figure 1:**
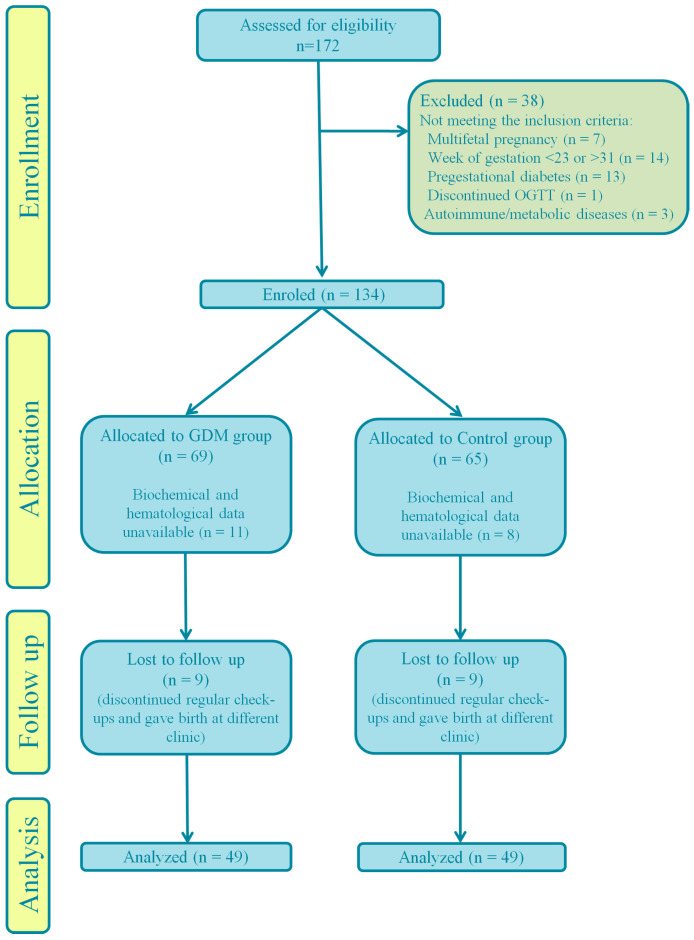
CONSORT flow diagram.

All the patients were at risk for GDM [15] and underwent oral glucose tolerance test (OGTT) with 75 g of glucose at the gestational age of 24–28 weeks. GDM was diagnosed according to criteria defined by the International Association of Diabetes and Pregnancy Study Groups (IADPSG): fasting plasma glucose ≥ 5.1 mmol/L, or 1-h OGTT glucose ≥ 10 mmol/L and 2-h OGTT glucose ≥ 8.5 mmol/L [16]. 172 patients underwent OGTT, but 93 had been included in the study (Figure 1).

Peripheral blood samples were obtained from GDM patients and normoglycemic controls in serum sampling tubes without anticoagulant and in sampling tubes with EDTA or sodium-citrate (for fibrinogen analysis) between pregnancy weeks 24 and 30, after an overnight fasting period. Data considered relevant for the present research included the diagnosis based on the results of OGTT, biochemical and hematological indicators of glucose (blood levels of glucose, insulin and glycated hemoglobin, (HbA1c)), and lipid status (concentrations of cholesterol, triglycerides, high- and low-density lipoproteins (HDL, LDL)), homeostatic model assessment (Homeostatic Model Assessment of Insulin Resistance (HOMA-IR) and β-cell function (HOMA-β)), iron and zinc metabolism (erythrocyte count (RBC), concentration of hemoglobin (Hb), mean corpuscular volume (MCV), mean corpuscular hemoglobin (MCH), mean corpuscular hemoglobin concentration (MCHC), serum iron, ferritin, total iron-binding capacity (TIBC), serum zinc concentration), liver function (transaminase activities), kidney function (levels of albumin, urea, creatinine and uric acid) and inflammatory status (C-reactive protein (CRP), sedimentation rate, leukocyte counts, fibrinogen). 

Hematological parameters were determined by using hematological analyzer Cell-Dyn Emerald (Abbott Diagnostics, Chicago, IL, USA). Basic biochemical parameters for the evaluation of liver and kidney function, CRP, HbA1c and lipid profile parameters were determined by employing commercial kits and by using Alinity I analyzer (Abbott Diagnostics, Chicago, IL, USA) and biochemical analyzer Bosystems A25 (BioSystems S.A., Barcelona, Spain). Insulin concentrations were measured by radioimmunoassay (RIA INSULIN (PEG), INEP, Belgrade, Serbia). Zinc concentration in sera samples was evaluated spectrophotometrically by using bromo-PAPS (Biosystems, Spain). HOMA-IR was calculated based on the equation HOMA-IR= (fasting insulin × fasting glucose)/22.5, while HOMA-β was computed as (20 × fasting insulin)/(fasting glucose − 3.5), since fasting insulin concentration was expressed in mU/L, while fasting glucose values were presented in mmol/L [17].

Study participants completed the survey in which they provided information about maternal age, height and self-reported pre-pregnancy weight (for the calculation of body mass index [BMI]), gestational age at blood sampling, gravidity and parity, and personal and family history of gestational or other types of diabetes, as well as data on administered therapy and supplementation.

Relevant information on pregnancy or delivery complications was obtained. Adverse pregnancy outcomes included spontaneous preterm labor (≤37 weeks), gestational hypertension, fetal growth disorders (macrosomia or fetal growth restriction), and oligohydramnios/polyhydramnios. Fetal macrosomia was defined as the term newborn birth weight ≥ 4000 g, and/or neonatal body weight > 90th ‰. Fetal growth restriction (FGR) was defined as neonatal body weight < 10th ‰. Oligohydramnios was defined as amniotic fluid index (AFI) ≤ 50, while polyhydramnios as AFI ≥ 240.

Data was also collected on newborn characteristics, including gestational age at birth, neonatal body weight and length, 1 min and 5 min Apgar score. The BMI of newborns was calculated using the same formula as for mothers, except for preterm infants, who were excluded from the correlation analyses related to this parameter.

Pregnancies with the presence of spontaneous preterm delivery with or without premature rupture of membranes, or fetal growth disturbances were considered to have adverse outcome, and both GDM and the control group were divided into subgroups: (a) with adverse outcome and (b) without adverse outcome (Table 1).

We compared all parameters between the groups (GDM and controls) and between the subgroups in each group (a) with adverse outcome and (b) without adverse outcome).

Statistical analysis of the obtained data was conducted by using the statistical software OriginPro 8.5.1 (OriginLab Corp., Northampton, MA, USA). The normality of the distribution of results was assessed by the Kolmogorov-Smirnov test, while the equality of variances in study groups was estimated by the F-test. For the paired comparison of normally distributed results, the two-tailed Student’s *t*-test was employed, while the Mann-Whitney U test was used to compare data that significantly deviated from normal distribution. The distributions of categorical variables in study groups were compared by the two-tailed chi-square test. A *p*-value of 0.05 was considered as a cutoff value for statistical significance. Results obtained by comparing the values of biochemical and hematological parameters between GDM patients and controls, as well as between subgroups of GDM patients stratified according to pregnancy outcome, are presented within box plots with displayed data points. The area under the curve (AUC) was calculated for the receiver-operating characteristic (ROC) curve constructed by using ROC plotter (https://www.rocplot.org/, assessed on 1 February 2024) [18] in order to determine the discriminatory ability of potential diagnostic and prognostic biomarkers.

## 3. Results

Data for women with GDM are listed in Table 2, while characteristics of healthy controls are presented in Table 3. Around 37% of patients diagnosed with GDM had pregnancy or delivery complications (Table 2), while in healthy controls the incidence of adverse pregnancy outcomes was 27% (Table 3). In the GDM group, basic characteristics of patients, such as age, smoking status, and gestational age at sampling, did not significantly differ between patients with and without adverse pregnancy outcome (Table 2). Furthermore, there were no significant differences in pre-pregnancy weight, BMI, and weight gain between these two subgroups of GDM patients. In the subgroup with adverse outcome, Cesarean section was more frequent; neonatal body weight, length, and BMI were higher; and Apgar score was lower (Table 2). The most frequent pregnancy/delivery complication in the GDM group was preterm labor (55.6%), followed by fetal macrosomia (27.7%). In a case with macrosomia shoulder dystocia occurred during delivery (Table 2). Similarly, in healthy controls, the average neonatal weight and BMI were significantly higher in the subgroup with adverse pregnancy outcome, while the average Apgar score at 1 min was lower. The most commonly detected complication in normoglycemic pregnancies was fetal macrosomia (58.3%) (Table 3). There were no cases with gestational hypertension or neonatal hypoglycemia. Neonatal hyperbilirubinemia with the need for phototherapy was present in 23 cases, with 11 in those with GDM and 12 in the control group.

**Figure 2:**
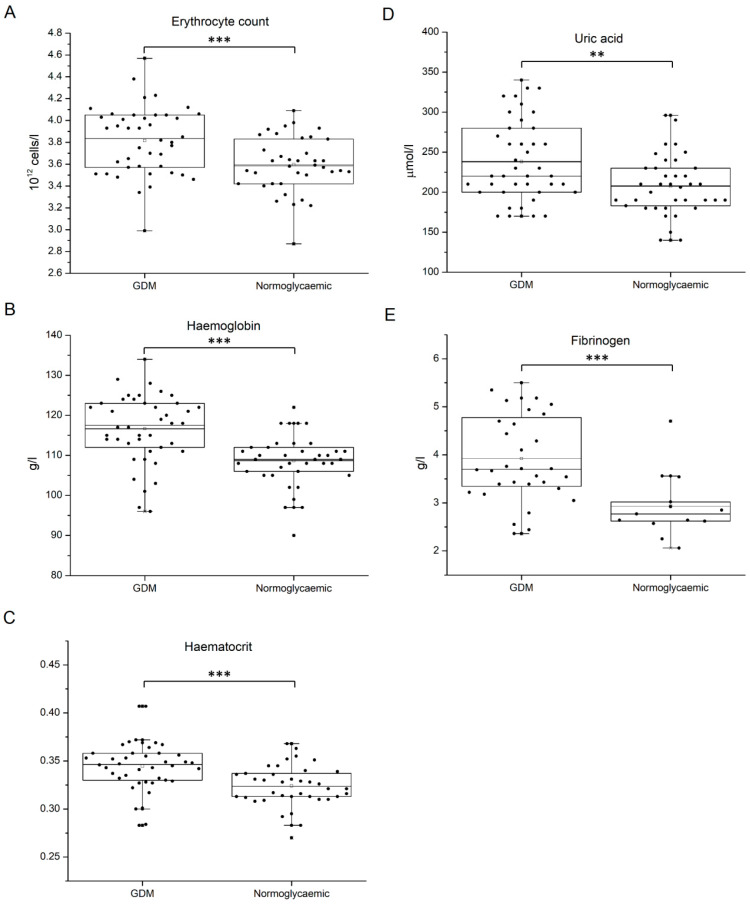
Differences in the values of biochemical parameters and the blood cell count between GDM patients and controls. (**A**) Erythrocyte count, (**B**) hemoglobin level, (**C**) hematocrit value, (**D**) uric acid level in serum, (**E**) plasma fibrinogen. Data are shown as interquartile range with median and mean value. Statistical significance was analyzed by Student’s *t*-test. *p* < 0.01 by two asterisks and *p* < 0.001 by three asterisks.

**Figure 3:**
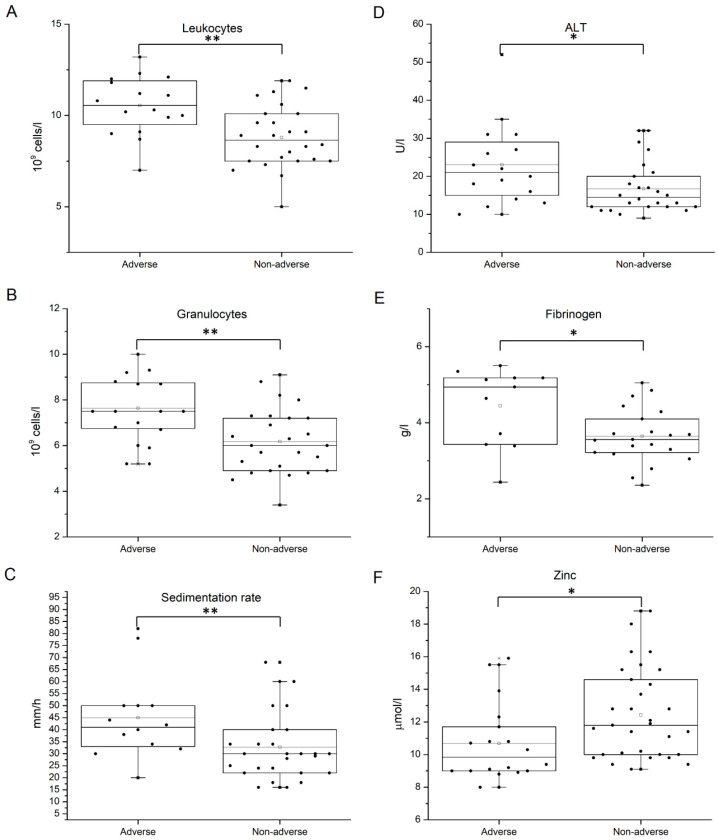
Differences in the values of biochemical parameters and the blood cell count between GDM patients with and without adverse pregnancy outcomes. (**A**) Leukocyte count, (**B**) granulocyte count, (**C**) sedimentation rate, (**D**) ALT, (**E**) plasma fibrinogen, (**F**) serum zinc level. Data are shown as interquartile range with median and mean value. Statistical significance was analyzed by Student’s *t*-test. *p* < 0.05 is indicated by one asterisk and *p* < 0.01 by two asterisks.

When biochemical characteristics and blood cell counts were compared between GDM patients and healthy controls, higher RBC, Hb concentration, and hematocrit (Hct) values were found in diabetic individuals (*p*-values < 0.001). Apart from erythrocyte-related parameters, as well as the fasting blood glucose concentration, increased levels of uric acid (*p* = 0.003) and fibrinogen (*p* < 0.001) were determined in GDM (Figure 2). Still, when these parameters associated with diagnosis were evaluated in GDM patients with and without adverse outcomes, statistically significant differences were found only for fibrinogen concentration (Table 4, Figure 3). Namely, in GDM patients with poor pregnancy outcome, fibrinogen level at blood sampling exhibited a significantly higher value than in other GDM patients (*p* = 0.016). Besides fibrinogen, ALT value, sedimentation rate, and granulocyte and total leukocyte counts were elevated in the poor-outcome GDM subgroup, while the serum level of zinc was significantly lower (Table 4, Figure 3).

In healthy pregnancies, the only biochemical parameter that differed between patients with and without poor pregnancy outcome was fibrinogen (Table 5). Similarly, as in GDM, a higher level of plasma fibrinogen was found in normoglycemic patients with adverse pregnancy outcomes (*p* = 0.002). Blood-cell-count-related parameters did not show statistically significant differences in the comparison between normoglycemic individuals with and without pregnancy/delivery complications (Table 5).

**Figure 4:**
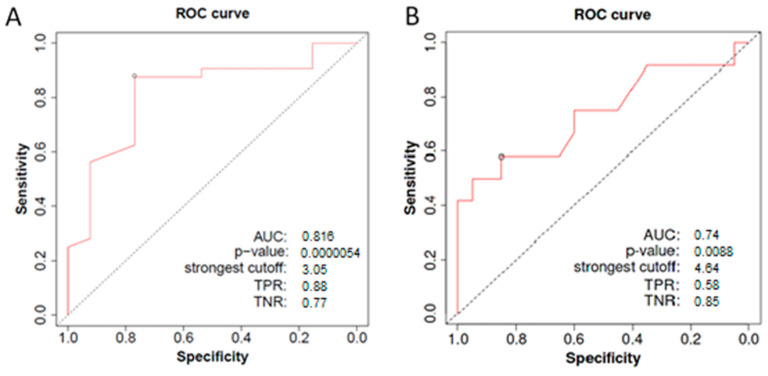
Receiver-operating characteristic curves (ROC) constructed to assess the ability of plasma fibrinogen level at week 24–30 to differentiate patients with GDM and normoglycemic controls (**A**), as well as GDM patients with and without poor pregnancy outcome (**B**). The corresponding AUC values, *p* values and the strongest cutoff values are presented for ROC curves. AUC—area under the curve, TPN—true positive rate, TNR—true negative rate.

Since fibrinogen was determined as the potential diagnostic and prognostic parameter in GDM, the ROC curve was constructed in order to assess its biomarker potential (Figure 4). The calculated AUC value for diagnostic ROC was 0.816 (*p* < 0.001, 95% CI 0.691–0.941), while the AUC value for the ROC curve designed for assessing the potential of fibrinogen to predict poor pregnancy outcome in GDM was 0.751 (*p* = 0.0096, 95% CI 0.561–0.941) (Figure 4).

## 4. Discussion

GDM represents a common pregnancy complication that may result in maternal and fetal/neonatal complications. Previous studies assessed different biomarkers as predictors and/or risk factors of GDM and were mostly focused on their diagnostic values when assessed in early pregnancy [13,14]. On the other hand, our present study focused on potential biomarkers as predictors of adverse pregnancy outcome at the time of OGTT performance, which could guide patient monitoring and/or intervention in high-risk GDM patients. Criteria for adverse outcome were selected to include major pregnancy and delivery complications associated with GDM, based on relevant literature. Gestational hypertension determined prior to OGTT and GDM diagnosis was among the exclusion criteria, since we aimed to avoid the inclusion of patients with hypertension unrelated to GDM, as well as an overlap of potential biomarkers of two independent pathophysiological mechanisms. Neonatal complications, such as hypoglycemia and hyperbilirubinemia, were not included as they may occur as independent complications with undefined etiology.

The evaluated pregnancy complications were more often seen in GDM. The most frequent pregnancy/delivery complication in the GDM group was preterm delivery, which may be explained by pathophysiology of GDM and preterm delivery which implies the involvement of (glycol)oxidative stress and inflammation [19]. The most commonly detected complication in normoglycemic patients was macrosomia. These results can be explained by the fact that OGTT was performed in normoglycemic patients because of their risk for GDM and fetal macrosomia. However, due to their normal OGTT result, they were not subjected to a strict diet and were less stringently monitored for glycemic control and lipid status at later stages of pregnancy. In line with the above—mentioned GDM managing regime, the lower incidence of macrosomia in GDM may result from carefully adjusted and strict dietary intervention after GDM had been diagnosed. 

By comparing GDM patients and healthy controls, higher RBC, Hb concentrations, Hct, and fasting glucose concentrations were found in diabetic individuals. Increased values of Hb, Hct, RBC, and fasting glucose concentrations in the early second trimester in patients with GDM have been reported by a previous cohort and cross-sectional studies, and a measurement oh Hbm Hct, RBC, and fasting glucose concentration was marked as a potential marker panel for early prediction of GDM [20,21,22]. Therefore, our results are in line with previous observations. 

Our study shows increased levels of uric acid (UA) in patients with GDM. Previous studies report that UA metabolism disturbance may potentially be associated with a more severe disturbance in glucose homeostasis by increasing insulin resistance and by suppressing insulin secretion [23]. Studies also report that higher levels of UA in GDM may be connected with adverse perinatal outcomes [24,25,26,27].

Levels of iron, ferritin and TIBC (total iron-binding capacity) were higher in patients with adverse outcome in the GDM group, although the difference was not statistically significant. The elevated serum ferritin might interact with other genetic and environmental factors, impairing β-cell functioning and affecting insulin secretion. Ferritin may have a direct role in the development of GDM responding to oxidative stress and producing superoxide that may mobilize stored iron from ferritin, increasing the pool of reactive iron and exacerbating oxidative stress. Elevated iron levels lead to oxidative stress in the body further aggravating insulin resistance and hyperglycemia. Ferritin is also an inflammation marker in the obese population [28]. Previous studies have reported increased iron and ferritin levels in GDM patients [29,30].

In addition to observations regarding iron-related parameters, our results demonstrated in zinc levels among patients with different outcomes in the GDM group, with lower levels observed in the adverse-outcome group. This finding aligns with the reported anti-inflammatory and anti-oxidation effects of zinc, for which reason zinc deficiency may be related to the increased inflammation and oxidative stress, which are associated with GDM-related pregnancy complications. Zinc may also be related to the maintenance of physiological glucose absorption, regulation of glucose utilization in cells, and reduction of the insulin resistance group. This metal ion is actively transported to the placenta, and zinc levels in umbilical cord correspond to the levels in maternal circulation. Placental transport of Zn may be altered in GDM, so fetal growth may be influenced by different mechanisms in GDM pregnancies. Lower zinc levels have been previously observed in GDM patients compared to normoglycemic patients [31,32,33]. Additionally, zinc supplementation in pregnant women is being investigated in the prevention of GDM [34].

Increased levels of fibrinogen were determined in GDM patients compared to normoglycemic controls, as well as in GDM patients with poor pregnancy outcome. There was an almost gradual increase in fibrinogen concentration from normoglycemic controls without adverse outcomes to GDM patients with adverse outcome. Fibrinogen, the largest plasma protein, increases during physiological pregnancy as a part of physiological hypercoagulability and reduced fibrinolytic activity. Furthermore, fibrinogen significantly increases as a response to tissue injury and inflammation, which is one of the hallmarks of hyperglycemic conditions. Previous studies report increased fibrinogen levels in midpregnancy in GDM compared with normoglycemic pregnancies, which supports our findings [26,35,36,37,38]. When it comes to pregnancy outcomes, altered biochemical parameters, such as low-density lipoprotein-cholesterol and fibrinogen are reported in small for gestational age infants born by mothers with GDM compared to normoglycemic mothers [39].

Apart from fibrinogen, other indicators of GDM-associated chronic low-level inflammation were elevated in GDM patients with poor pregnancy outcomes, including leukocyte and granulocyte counts, ALT, and sedimentation rate, supporting the involvement of this process in the pathophysiology of GDM-related complications. This observation further implies the potential beneficiary effect of antioxidants and anti-inflammatory therapeutics in GDM pregnancies. 

Pathophysiology of GDM includes dysregulated mechanisms of gluconeogenesis; chronic inflammation; and oxidative stress associated with the involvement of different placental signaling molecules and stimulated by genetic, epigenetic and environmental factors. It has been proven that different biomolecules are altered in GDM pregnancies, with a relatively high potential to enable early prediction of GDM [7]. In GDM pregnancies resulting in an adverse outcome, there is a possibility that these mechanisms are enhanced. Therefore, these potential biological markers of GDM are expected to be additionally altered in those pregnancies, so their determination during mid-pregnancy at the time of OGTT may predict potential adverse pregnancy outcome. 

We conducted this research as a pilot study in order to test the hypothesis whether biomarkers may be used as predictors of adverse pregnancy outcome in GDM patients in order to conduct intensive monitoring and we identified fibrinogen as the best diagnostic and prognostic parameter in GDM among the tested potential markers. The main limitation of our study is a relatively small sample size, and this study may be considered as a pilot study that is an introduction to a future large multicenter study. 

## 5. Conclusions

Concerning the fact that prevalence of GDM is dramatically increasing worldwide with short- and long-term consequences for maternal and fetal/neonatal/infant health, there is a need for the identification of biomarkers that can be used for the prediction of adverse pregnancy outcome in GDM patients and indicate intensive monitoring of those pregnancies. The results of our study identified fibrinogen as a parameter with both diagnostic and prognostic ability in GDM. Furthermore, inflammation-related parameters demonstrated prognostic potential in our study group. The results are promising in terms of the identification of potential biomarkers useful for the prediction of adverse pregnancy outcome, and future multicenter studies are needed in order to confirm and expand our results.

## Figures and Tables

**Table 1:** Inclusion and exclusion criteria and criteria for the adverse outcome.

Inclusion Criteria	Exclusion Criteria	Adverse Outcome
Indication for OGTT	Multiple pregnancies	Preterm delivery
Gestational age—24–30 weeks	Gestational age < 23 or >31 weeks	Fetal growth disturbances (macrosomia or FGR)
Singleton pregnancy	Metabolic disease	Polyhydramnios
	Hypertension	Oligohydramnios
	Fetal anomaly	Shoulder dystocia

**Table 2:** Characteristics of GDM patients.

	With Adverse Outcome N = 18	Without Adverse Outcome N = 31	*p*
Age (years) ^a^	33.4 ± 3.6	35.3 ± 4.3	0.11
Smoking status (%)	11.7	29	0.27
Gestational age at sampling (weeks)	27.2 ± 2.1	27.16 ± 2.0	0.99
Gravidity, n (%)			
1	9 (50)	7 (22.6)	0.09
2	6 (33.3)	11 (35.5)	
≥3	3 (16.7)	13 (41.9)	
Parity, n (%)			
1	12 (66.7)	11 (35.5)	0.11
2	4 (22.2)	14 (45.2)	
≥3	2 (11.1)	6 (19.3)	
Family history of diabetes (%)	47	31	0.28
Pre-pregnancy weight (kg) ^a^	70.6 ± 15.6	71.1 ± 12.4	0.91
Height (cm) ^a^	167.6 ± 6.5	169.1 ± 4.8	0.36
Weight gain (kg) ^a^	6.8 ± 7.5	7.5 ± 4.0	0.70
Pre-pregnancy BMI	25.1 ± 5.6	24.9 ± 4.6	0.88
*Delivery, newborn characteristics and obstetric complications* ^a^			
Cesarean section, n (%)	13 (72.2)	8 (25.8)	**0.005**
Weight (g) ^b^	3900.0 ± 596.2	3263.5 ± 313.3	<0.001 *
Length (cm) ^b^	53.0 ± 2.4	50.9 ± 1.5	**0.005**
BMI ^b^	1.11 ± 0.25	0.85 ± 0.13	**<0.001**
Apgar score at 1 min	8.67 ± 0.74	9 ± 0.25	**0.03**
Apgar score at 5 min	9.55 ± 0.76	9.93 ± 0.24	**0.016**
Preterm labor, n (%)	10 (55.6)	-	-
Macrosomia, n (%)	5 (27.8)	-	-
FGR, n (%)	2 (11.1)	-	-
Polyhydramnios, n (%)	2 (11.1)	-	-

^a^ Mean ± SD, ^b^ preterm infants not included, * statistically significant results are shown in bold Abbreviations: GDM—gestational diabetes mellitus; BMI—body mass index; FGR—fetal growth restriction.

**Table 3:** Characteristics of normoglycemic patients.

	With Adverse Outcome N = 12	Without Adverse Outcome N = 32	*p*
Age (years) ^a^	32.8 ± 4.9	31.8 ± 3.9	0.50
Smoking status (%)	16.7	21.9	0.70
Gestational age at sampling (weeks)	25.5 ± 1.7	27.1 ± 1.6	**0.009**
Gravidity, n (%)			
1	2 (16.7)	11 (34.4)	0.32
2	7 (58.3)	11 (34.4)	
≥3	3 (25)	10 (31.2)	
Parity, n (%)			
1	5 (41.7)	16 (50)	0.60
2	6 (50.0)	11 (34.4)	
≥3	1 (8.3)	5 (15.6)	
Family history of diabetes (%)	16.7	12.5	0.87
Pre-pregnancy weight (kg) ^a^	65.7 ± 9.9	67.1 ± 7.4	0.62
Height (cm) ^a^	171.3 ± 7.2	172.2 ± 5.9	0.67
Weight gain (kg) ^a^	7.6 ± 2.4	7.9 ± 3.6	0.80
Pre-pregnancy BMI	22.3 ± 3.0	22.6 ± 2.7	0.74
*Delivery, newborn characteristics and obstetric complications* ^a^			
Cesarean section, n (%)	5 (41.7)	8 (25)	**0.005**
Weight (g) ^b^	3859.0 ± 794.1	3438.4 ± 323.7	**0.023**
Length (cm) ^b^	53.3 ± 2.9	51.9 ± 1.5	0.057
BMI ^b^	1.12 ± 0.32	0.93 ± 0.14	**0.012**
Apgar score at 1 min	8.75 ± 0.43	9.00 ± 0.25	**0.025**
Apgar score at 5 min	9.75 ± 0.43	9.94 ± 0.24	0.08
Preterm labor, n (%)	2 (16.7)	-	-
Macrosomia, n (%)	7 (58.3)	-	-
FGR, n (%)	2 (16.7)	-	-
Polyhydramnios, n (%)	1 (8.3)	-	-

^a^ Mean ± SD, ^b^ preterm infants not included, statistically significant results are shown in bold Abbreviations: GDM—gestational diabetes mellitus; BMI—body mass index; FGR—fetal growth restriction.

**Table 4:** Biochemical parameters and blood cell counts of GDM patients.

	With Adverse Outcome N = 18	Without Adverse Outcome N = 31	*p*
*Glycemic status* ^a^			
OGTT (mmol/L)			
0′	4.83 ± 0.46	4.86 ± 0.78	0.85
60′	10.74 ± 1.83	10.90 ± 1.50	0.74
120′	8.72 ± 1.55	9.00 ± 2.34	0.66
Fasting insulin (mU/L)	23.32 ± 17.16	15.53 ± 6.13	0.76
HOMA-IR	5.04 ± 5.13	3.46 ± 1.75	0.70
HOMA-β	3.86 ± 4.86	2.66 ± 1.78	0.53
HbA1c (%)	4.79 ± 0.15	4.75 ± 0.24	0.64
*Lipid profile* ^a^			
Triglycerides (TG) (mmol/L)	2.45 ± 0.81	2.36 ± 0.82	0.76
Cholesterol (mmol/L)	7.30 ± 1.36	6.92 ± 1.82	0.53
HDL (mmol/L)	1.98 ± 0.43	1.92 ± 0.45	0.68
LDL (mmol/L)	4.28 ± 1.12	3.90 ± 1.86	0.51
TG/HDL	1.34 ± 0.58	1.31 ± 0.65	0.89
LDL/HDL	2.25 ± 0.72	2.16 ± 1.53	0.85
*Other biochemical parameters* ^a^			
Total proteins (g/L)	64.12 ± 3.64	64.08 ± 2.91	0.96
Albumin (g/L)	35.13 ± 1.77	35.25 ± 2.07	0.86
Urea (mmol/L)	3.29 ± 0.92	3.13 ± 0.89	0.59
Creatinine (µmol/L)	52.12 ± 8.81	50.08 ± 6.26	0.40
Uric acid (µmol/L)	235.00 ± 47.17	240.00 ± 52.33	0.76
CRP (mg/L)	9.77 ± 13.09	6.26 ± 5.08	0.58
AST (U/L)	18.19 ± 4.90	16.69 ± 5.45	0.39
ALT (U/L)	23.06 ± 10.33	16.73 ± 6.62	**0.023**
Fibrinogen (g/L)	4.44 ± 0.98	3.65 ± 0.70	**0.016**
Iron (µmol/L)	17.13 ± 5.66	14.25 ± 4.56	0.10
Ferritin (µg/L)	22.38 ± 8.53	18.31 ± 13.08	0.39
TIBC (µmol/L)	62.32 ± 10.77	65.85 ± 10.96	0.29
Zinc (µmol/L)	10.68 ± 2.28	12.42 ± 2.66	**0.027**
*Complete blood count* ^a^			
Erythrocytes (10^12^ cells/L)	3.80 ± 0.34	3.82 ± 0.28	0.83
Hemoglobin (g/L)	115.50 ± 9.95	117.38 ± 7.26	0.49
Hematocrit	0.344 ± 0.029	0.345 ± 0.023	0.96
Sedimentation rate (mm/h)	45.00 ± 17.64	32.74 ± 13.69	**0.028**
MCV (fl)	90.64 ± 2.93	90.30 ± 4.81	0.80
MCH (pg/cell)	30.41 ± 1.30	30.77 ± 1.82	0.51
MCHC (g/L)	335.25 ± 7.75	340.73 ± 9.27	0.06
Leukocytes (10^9^ cells/L)	10.54 ± 1.55	8.79 ± 1.64	**0.002**
Thrombocytes (10^9^ cells/L)	253.00 ± 52.01	220.46 ± 51.52	0.06
Granulocytes (10^9^ cells/L)	7.64 ± 1.32	6.17 ± 1.40	**0.002**
Lymphocytes (10^9^ cells/L)	2.20 ± 0.36	2.04 ± 0.31	0.15

^a^ Mean ± SD, statistically significant results are shown in bold. Abbreviations: GDM—gestational diabetes mellitus; OGTT—oral glucose tolerance test; HOMA-IR—homeostatic model assessment of insulin resistance; HOMA-β—homeostasis model assessment of β-cell function; HbA1c—glycated hemoglobin; TG—triglycerides; HDL—high-density lipoprotein; LDL—low-density lipoprotein; CRP—C-reactive protein; AST—aspartate aminotransferase; ALT—alanine aminotransferase; TIBC—total iron-binding capacity; MCV—mean corpuscular volume; MCH—mean corpuscular hemoglobin; MCHC—mean corpuscular hemoglobin concentration.

**Table 5:** Biochemical parameters and blood cell counts of normoglycemic patients.

	With Adverse Outcome N = 12	Without Adverse Outcome N = 32	*p*
*Glycemic status* ^a^			
OGTT (mmol/L)			
0′	4.39 ± 0.31	4.38 ± 0.38	0.93
60′	8.04 ± 1.27	7.26 ± 1.14	0.06
120′	6.86 ± 0.76	6.55 ± 1.08	0.38
Fasting insulin (mU/L)	15.78 ± 6.66	16.46 ± 10.59	0.68
HOMA-IR	3.08 ± 1.38	3.26 ± 2.20	0.56
HOMA-β	4.51 ± 3.52	4.56 ± 4.21	0.89
HbA1c (%)	4.69 ± 0.21	4.70 ± 0.29	0.86
*Lipid profile* ^a^			
Triglycerides (TG) (mmol/L)	2.21 ± 0.67	2.20 ± 0.62	0.95
Cholesterol (mmol/L)	7.22 ± 1.05	6.74 ± 0.80	0.15
HDL (mmol/L)	2.31 ± 0.44	2.05 ± 0.46	0.10
LDL (mmol/L)	3.87 ± 1.13	3.73 ± 0.80	0.67
TG/HDL	1.06 ± 0.72	1.18 ± 0.68	0.66
LDL/HDL	1.72 ± 0.56	1.90 ± 0.78	0.51
*Other biochemical parameters* ^a^			
Total proteins (g/L)	65.78 ± 3.32	63.93 ± 3.68	0.20
Albumin (g/L)	35.81 ± 2.80	35.82 ± 2.21	0.99
Urea (mmol/L)	2.73 ± 0.52	2.84 ± 0.93	0.76
Creatinine (µmol/L)	50.11 ± 5.86	53.14 ± 6.58	0.24
Uric acid (µmol/L)	221.11 ± 38.71	203.55 ± 33.82	0.21
CRP (mg/L)	5.34 ± 3.45	5.21 ± 3.10	0.96
AST (U/L)	17.14 ± 6.92	17.54 ± 5.50	0.88
ALT (U/L)	18.14 ± 12.84	14.86 ± 7.29	0.42
Fibrinogen (g/L)	3.68 ± 0.64	2.60 ± 0.28	**0.002**
Iron (µmol/L)	14.66 ± 6.77	14.82 ± 6.66	0.95
Ferritin (µg/L)	17.24 ± 13.06	13.74 ± 9.49	0.46
TIBC (µmol/L)	61.10 ± 11.10	61.32 ± 11.00	0.84
Zinc (µmol/L)	13.18 ± 3.25	11.29 ± 2.78	0.07
*Complete blood count* ^a^			
Erythrocytes (10^12^ cells/L)	3.72 ± 0.21	3.56 ± 0.25	0.09
Hemoglobin (g/L)	112.00 ± 5.60	107.62 ± 6.11	0.07
Hematocrit	0.332 ± 0.022	0.322 ± 0.020	0.20
Sedimentation rate (mm/h)	36.60 ± 15.57	32.43 ± 10.98	0.38
MCV (fl)	89.19 ± 1.97	90.98 ± 6.22	0.41
MCH (pg/cell)	30.14 ± 0.66	30.38 ± 2.07	0.75
MCHC (g/L)	338.00 ± 8.54	335.00 ± 7.47	0.33
Leukocytes (10^9^ cells/L)	9.06 ± 1.77	9.24 ± 1.66	0.78
Thrombocytes (10^9^ cells/L)	257.33 ± 52.80	236.34 ± 43.77	0.25
Granulocytes (10^9^ cells/L)	6.59 ± 1.60	6.61 ± 1.39	0.96
Lymphocytes (10^9^ cells/L)	1.80 ± 0.27	1.99 ± 0.43	0.25

^a^ Mean ± SD, statistically significant results are shown in bold. Abbreviations: OGTT—oral glucose tolerance test; HOMA-IR—homeostatic model assessment of insulin resistance; HOMA-β—homeostasis model assessment of β-cell function; HbA1c—glycated hemoglobin; TG—triglycerides; HDL—high-density lipoprotein; LDL—low-density lipoprotein; CRP—C-reactive protein; AST—aspartate aminotransferase; ALT—alanine aminotransferase; TIBC—total iron-binding capacity; MCV—mean corpuscular volume; MCH—mean corpuscular hemoglobin; MCHC—mean corpuscular hemoglobin concentration.

## Data Availability

The data presented in this study are available on request from the corresponding author. The data are not publicly available due to ethical regulations.
